# Umbilical cord mesenchymal stromal cells in serum-free defined medium display an improved safety profile

**DOI:** 10.1186/s13287-023-03604-0

**Published:** 2023-12-12

**Authors:** Xiaoyun Wu, Zhijie Ma, Yuxiao Yang, Yongxu Mu, Daocheng Wu

**Affiliations:** 1 Key Laboratory of Biomedical Information Engineering of the Ministry of Education, School of Life Science and Technology, Xi’an Jiaotong University, Xi’an, 710049 People’s Republic of China; 2 Interventional Department, The First Affiliated Hospital of Baotou Medical College, Inner Mongolia University of Science and Technology, Baotou, Inner Mongolia People’s Republic of China; 3 Department of Pharmacy, Beijing Friendship Hospital, Capital Medical University, Beijing, People’s Republic of China; 4 Department of Technology, Beijing Stem Cell (ProterCell) Biotechnology Co., Ltd., Beijing, People’s Republic of China; 5 Department of Technology, Inner Mongolia Stem Cell (ProterCell) Biotechnology Co., Ltd., Hohhot, People’s Republic of China; 6 Department of Technology, Research Center for Hua-Da Precision Medicine of Inner Mongolia Autonomous Region, Hohhot, People’s Republic of China

**Keywords:** Immunogenicity, Mesenchymal stromal cells, Serum free, Safety, Tumorigenicity, Umbilical cord

## Abstract

**Background:**

Safety evaluations in preclinical studies are needed to confirm before translating a cell-based product into clinical application. We previously developed a serum-free, xeno-free, and chemically defined media (S&XFM–CD) for the derivation of clinical-grade umbilical cord-derived MSCs (UCMSCs), and demonstrated that intraperitoneal administration of UCMSCs in S&XFM–CD (UCMSC^S&XFM−CD^) exhibited better therapeutic effects than UCMSCs in serum-containing media (SCM, UCMSC^SCM^). However, a comprehensive investigation of the safety of intraperitoneal UCMSC^S&XFM−CD^ treatment should be performed before clinical applications.

**Methods:**

In this study, the toxicity, immunogenicity and biodistribution of intraperitoneally transplanted UCMSC^S&XFM−CD^ were compared with UCMSC^SCM^ in rats via general vital signs, blood routine, blood biochemistry, subsets of T cells, serum cytokines, pathology of vital organs, antibody production and the expression of human-specific gene. The tumorigenicity and tumor-promoting effect of UCMSC^S&XFM−CD^ were compared with UCMSC^SCM^ in nude mice.

**Results:**

We confirmed that intraperitoneally transplanted UCMSC^S&XFM−CD^ or UCMSC^SCM^ did not cause significant changes in body weight, temperature, systolic blood pressure, diastolic blood pressure, heart rate, blood routine, T lymphocyte subsets, and serum cytokines, and had no obvious histopathology change on experimental rats. UCMSC^S&XFM−CD^ did not produce antibodies, while UCMSC^SCM^ had very high chance of antibody production to bovine serum albumin (80%) and apolipoprotein B-100 (60%). Furthermore, intraperitoneally injected UCMSC^S&XFM−CD^ were less likely to be blocked by the lungs and migrated more easily to the kidneys and colon tissue than UCMSC^SCM^. In addition, UCMSC^S&XFM−CD^ or UCMSC^SCM^ showed no obvious tumorigenic activity. Finally, UCMSC^S&XFM−CD^ extended the time of tumor formation of KM12SM cells, and decreased tumor incidence than that of UCMSC^SCM^.

**Conclusions:**

Taken together, our data indicate that UCMSC^S&XFM−CD^ display an improved safety performance and are encouraged to use in future clinical trials.

**Supplementary Information:**

The online version contains supplementary material available at 10.1186/s13287-023-03604-0.

## Introduction

Umbilical cord (UC) tissue is easily available and is thus a useful source of mesenchymal stromal cells (MSCs) [1, 2]. UC-derived MSCs (UCMSCs) show no significant variations between donors as they are obtained from newborn infants, thus lacking age-associated differences [3] and suggesting their suitability for cell-based treatments. Positive results have been obtained on the use of USMSCs in different in vivo studies, suggesting their potential for clinical applications [4]. However, questions have been raised on their safety, immunogenicity, biodistribution, and oncological implications in cases of previous malignancy, as well as the use of allogeneic MSCs [5].

The UCMSCs used in preclinical trials are usually grown in media containing fetal bovine serum (FBS), in line with the preparation of clinical-grade MSCs [6, 7]. However, the use of FBS, an animal-derived product, has raised serious safety concerns associated with the potential transmission of unknown viruses and the induction of undesirable immunological reactions [8]. A further issue is that FBS is poorly defined, presenting difficulties for standardization, which may adversely affect the clinical application of MSCs. There is also increasing concern regarding the regulation of animal sera in cell culture materials [9, 10]. In China, the quality control guidelines for the preparation of stem cells specify that animal sera should be avoided as far as possible, and encourage the use of serum-free media instead of serum-containing media (SCM) during the preparation of stem cells for clinical applications [11, 12]. In this regard, several studies have described commercially available serum-free media that can be used for isolating and growing MSCs (listed in Table 1 in our previous article [13]). However, these disclosed or commercial defined media cannot support large-scale expansion of UCMSCs in our laboratory. We have previously developed a serum-free, xeno-free, and chemically defined medium (S&XFM–CD) for the derivation of clinical-grade UCMSCs that contains nutrients, hormones, minerals, and growth factors (see Patent No. CN. ZL201210350602.0 and [13]). A previous study confirmed the superiority of delivery by intraperitoneal, rather than intravenous or anal injection, showing higher numbers of MSCs after delivery and better recovery from experimental colitis, suggesting that this might be the best route for delivering MSCs during colitis treatment [14]. Furthermore, we demonstrated that intraperitoneal delivery of UCMSCs in S&XFM–CD medium (UCMSC^S&XFM−CD^) was therapeutically more effective than the administration of UCMSCs in SCM (UCMSC^SCM^), shown by the enhancement of macrophage polarization from the proinflammatory M1 to anti-inflammatory M2 stages in an experimental model of acute colitis [15]. Furthermore, no transformation was observed in UCMSC^S&XFM−CD^ and both the normal karyotype and genome were preserved, allowing their successful long-term culture in vitro [13]. However, the safety of using UCMSCs in serum-free medium remains debatable due to infrequent safety analyses and a lack of comprehensive tests. In addition, the safety of intraperitoneal delivery of UCMSC^S&XFM−CD^ requires in-depth evaluation before this strategy can be translated into clinical application. Safety is critical for clinical applications of cell-based products [2], and the safety of these products thus need to be thoroughly evaluated in both preclinical and in vitro assessments [16].

**Table 1:** Vital sign assessment

Parameters	Day 14			Day 35		
	PBS	UCMSC^SCM^	UCMSC^S&XFM−CD^	PBS	UCMSC^SCM^	UCMSC^S&XFM−CD^
Weight (g)	279.75 ± 55.15	281.38 ± 53.40	279.63 ± 58.08	303.63 ± 63.59	308.25 ± 66.30	309.38 ± 67.94
Temperature (°C)	38.08 ± 0.23	38.26 ± 0.30	38.36 ± 0.35	38.1625 ± 0.41	38.3 ± 0.30	38.2625 ± 0.23
SBP (mmHg)	107.42 ± 6.22	114.22 ± 6.95	109.10 ± 11.52	108.69 ± 8.20	113.36 ± 5.18	115.71 ± 5.08
DBP (mmHg)	73.27 ± 4.05	77.33 ± 6.12	74.36 ± 7.07	74.81 ± 7.02	76.96 ± 4.36	79.33 ± 4.16
HR (BPM)	379.22 ± 35.90	377.72 ± 26.91	369.17 ± 23.11	373.00 ± 32.10	370.50 ± 28.59	380.22 ± 11.41

Data are expressed as mean ± SD

*SBP* systolic blood pressure, *DBP* diastolic blood pressure, *HR* heart rate, *BPM* beats per minute

Compared with the PBS group,* P* ≥ 0.05, compared with the UCMSC^SCM^ group, *P* ≥ 0.05

Potential adverse events related to MSC use, including the risk of biodistributed, immunological, and oncological safety concerns, have received increasing attention in the last decade [17]. In addition, differences in cell culture condition may affect MSC characteristics, which may influence their safety, biological actions, and therapeutic effectiveness [18]. Biosafety analyses related to biodistribution, tumor-promoting effects, and the immunological consequences of UCMSC^S&XFM−CD^ and UCMSC^SCM^ administration require further comparison and evaluation. Hence, in this study, we compared the differences in safety-related biological characteristics between UCMSC^S&XFM−CD^ and UCMSC^SCM^ such as toxicity, immunogenicity, biodistribution, tumorigenicity, and tumor-promoting effects, and comprehensively evaluated the biosafety of UCMSC^S&XFM−CD^.

## Materials and methods

### UCMSCs isolation

Human umbilical cord tissue was obtained at the First Affiliated Hospital of Baotou Medical College from a full-term healthy infant delivered after a cesarean section, whose mother had signed an informed consent form and the procedure was approved by the Medical Ethics Committee of Baotou Medical College (No: 2021-033). Isolation and culture of UCMSCs were performed as previously described [19]. Briefly, the UC was rinsed with sterile phosphate-buffered saline (PBS) to remove any remaining blood, and the vessels were separated from the cord segments. After thorough washing with PBS, Wharton’s jelly was initially chopped and then sliced into pieces (0.5‒1 mm) which, following removal of the cord segments, were incubated for five days in either S&XFM–CD or SCM (IMDM with 10% FBS) at 37 °C and 5% CO_2_. Approximately half of the culture medium was replaced every two to three days. UCMSCs were passaged when 80‒90% confluent to densities of 3000 cells/cm^2^.

### Flow cytometry

UCMSC^S&XFM−CD^ and UCMSC^SCM^ from the fifth passage were harvested, resuspended as single-cell suspensions, and incubated (1 h, 4 °C, in the dark) with commercial antibodies against CD14, CD19, CD29, CD34, CD44, CD45, CD73, CD90, CD105, and HLA-DR. The antibody details are provided in Additional file 1: Table S1 (all reagents were from BD). A FACSCalibur flow cytometer (Becton Dickinson, Franklin Lakes, NJ, USA) was used with data analysis with CellQuest Pro 3.7 software (Becton Dickinson).

In the animal experiments, blood samples were taken from the orbital venous plexus of the rats. After isolation, mononuclear cells were incubated as above with commercial antibodies against CD3, CD4, and CD8 (all reagents were from BD, Additional file 1: Table S1). The proportions of CD3^+^, CD4^+^, and CD8^+^ T cells, and the CD4^+^/CD8^+^ T cell ratios were analyzed on the flow cytometer as above.

### Lineage differentiation and staining

UCMSC^S&XFM−CD^ and UCMSC^SCM^ at passage 5 were differentiated into adipocytes, osteocytes, and chondrocytes. Histochemistry was performed as described previously [20] (All reagents were from Cyagen, Guangzhou, China).

### Animal experiments

All animals were provided and raised by the Experimental Animal Center of the First Affiliated Hospital of Baotou Medical College, with 12-h light–dark cycle and free access to food and water. All animal operations were approved by the Ethics Committee of Baotou Medical College (No: 2021-033) and followed the guidelines for the care and use of experimental animals of the National Institutes of Health. Then the animals were euthanized by intraperitoneal injection of pentobarbital sodium (180 mg/kg), and the follow-up experiments were carried out.

### Toxicity, immunogenicity, and biodistribution assays

15 six-week-old male Sprague–Dawley (SD) rats (weighing 200 ± 20 g) were randomly divided into three groups, with 5 rats in each group. Of these, the UCMSC^S&XFM−CD^ and UCMSC^SCM^ groups received respective doses of UCMSC^S&XFM−CD^ and UCMSC^SCM^ (2 × 10^6^ cells/kg body weight) suspended in 0.5 mL PBS via intraperitoneal injection once at the beginning of the study, and the PBS group received 0.5 mL of PBS.

### Antibody detection experiments

Rats received intraperitoneal injections of UCMSC^S&XFM−CD^ or UCMSC^SCM^ (2 × 10^6^ cells/kg body weight) in 0.5 ml of PBS (n = 5/group). Further injections were given weekly for the subsequent three weeks. Blood samples for antibodies against bovine serum albumin (BSA) and apolipoprotein B-100 (apoB-100) were collected before the injections and one week following the last injection.

### Tumorigenicity assay

20 six-week-old male nude mice (weighing 20 ± 2 g) were randomly assigned to three groups, with 5 mice in each group. The groups received single subcutaneous inoculations of UCMSCS^&XFM−CD^, UCMSC^SCM^, and human colon cancer KM12SM cells (2 × 10^6^ cells/mouse), respectively, into the lateral trunk region. The mice were checked twice a week thereafter for tumor growth (for 3 months).

### Tumor-promoting assay

Nude mice as above were randomly divided into three groups. Single subcutaneous inoculations of KM12SM cells (2 × 10^6^ cells/mouse) were given into the lateral trunk region alone or in conjunction with UCMSC^S&XFM−CD^ or UCMSC^SCM^ at a 1:1 ratio. After inoculation, the mice were observed twice a week for tumor growth. After sacrifice, the tumors were weighed and the volumes were calculated as ½ × minor axis^2^ × major axis.

### General vital sign detection

The vital signs of the rats, including anal temperature, weight, blood pressure, and heart rate were observed and recorded. The injection sites were checked, anal temperatures were measured with thermometer (Ji Nuo Tai Technology, Beijing, China), and the blood pressure and heart rates with a Softron 2006A meter (Softron Biotechnology, Beijing, China).

### Blood routine and biochemical index detection

Blood samples were collected from orbital venous plexus, and performed to detect and compare blood routine and biochemical indexes by using the kits (Jiancheng, Nanjing, China).

### Enzyme-linked immunosorbent assays

Blood was collected and assayed for antibodies against BSA and apoB-100 by ELISA (Absin, Shanghai, China) as previously described [8, 21]. Interferon (IFN)-γ, tumor necrosis factor (TNF)-α, interleukin (IL)-4, and IL-6 levels in serum were also measured by ELISA (Neobioscience, Shenzhen, China).

### Histological analysis

Fixed tissues (lung, liver, kidney, heart, testis, and colon) were paraffin-embedded, sectioned (5 µm), and stained with hematoxylin and eosin. The stained sections were observed by a blinded histopathologist under light microscopy to check for pathological changes.

### qRT-PCR

Standard curves for the assessment of the biodistribution of UCMSC^S&XFM−CD^ and UCMSC^SCM^ were generated by administering serial dilutions of UCMSCs to mouse tissues. UCMSCs (2 × 10, 2 × 10^2^, 2 × 10^3^, 2 × 10^4^, or 2 × 10^5^) were added to the whole mouse tissues prior to homogenization. Total RNA was extracted from the hearts, brains, lungs, livers, kidneys, spleens, quadriceps, and iliac marrow of the rats using TRIzol reagent (Invitrogen, USA) and reverse-transcribed to cDNA using the QuantiTect Reverse Transcription Kit (Qiagen, Germany). The cDNA was amplified by qRT-PCR using Platinum SYBR Green PCR Mix (Invitrogen, USA) and a 7700 Sequence Detector (Applied Biosystems, USA) with human and mouse GAPDH primers (Human GAPDH primers: Forward primer, TGC TTT TAA CTC TGG TAA AGT GGA TA; Reverse primer, GTG GAA TCA TAT TGG AAC ATG TAA AC. Mouse GAPDH primers: forward primer, CAG CGA CAC CCA CTC CTC CAC CTT; reverse primer, CAT GAG GTC CAC CAC CCT GTT GCT).

### Statistical analysis

The data were presented as mean ± standard deviation (SD). Differences between two groups were analyzed by t-tests and those between multiple groups by one-way analysis of variance (ANOVA) followed by Tukey’s multiple comparisons tests. *P* < 0.05 was considered statistically significant. Data were analyzed using SPSS 17.0 (SPSS, IL, USA).

## Results

### Characterization of UCMSCS^&XFM−CD^ and UCMSC^SCM^

**Fig. 1:**
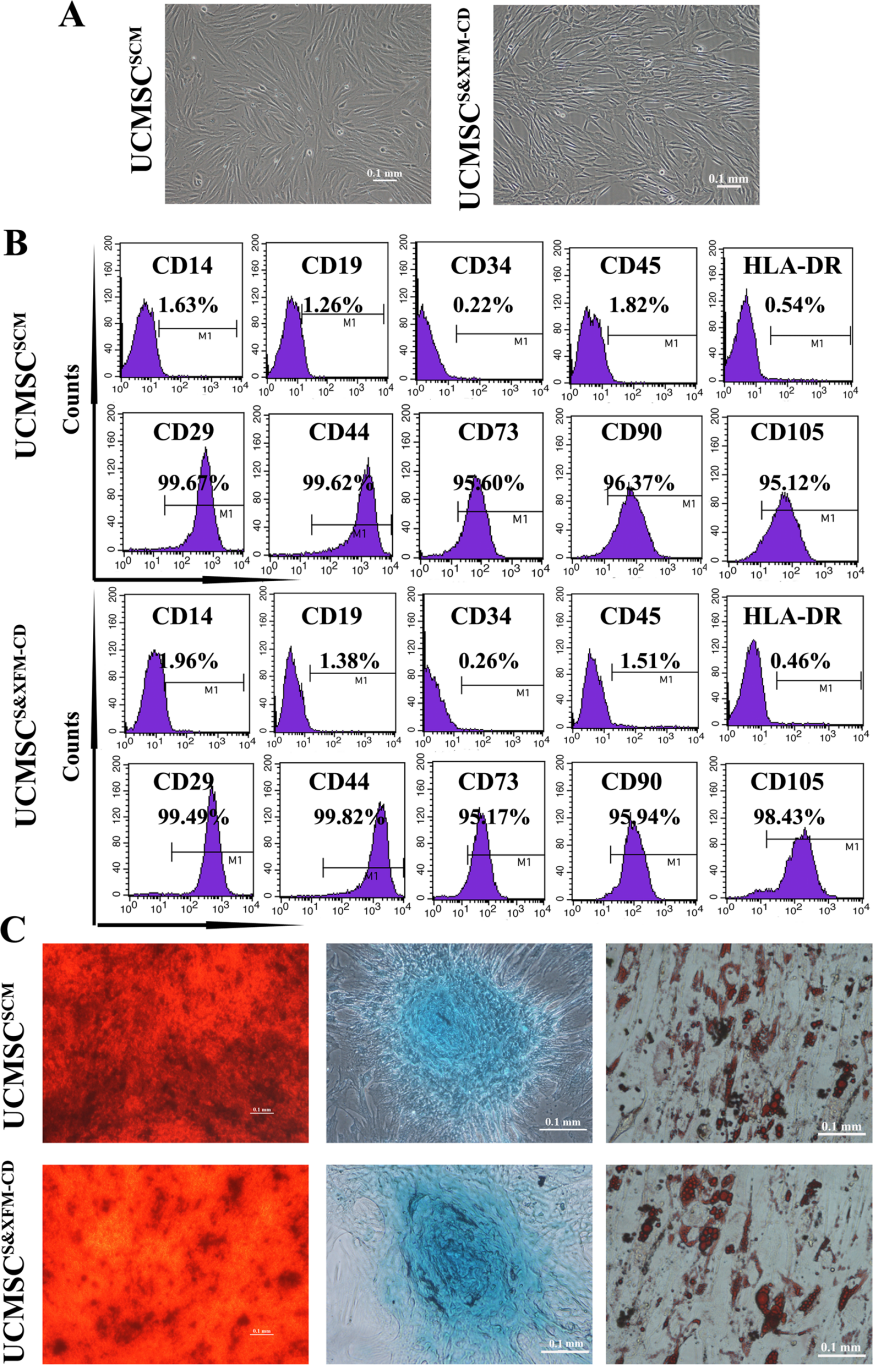
The characterization analysis of UCMSC^S&XFM−CD^ and UCMSC^SCM^. **A** Morphology characteristics of UCMSC^S&XFM−CD^ and UCMSC^SCM^. Scale bar = 100 μm. **B** Flow cytometry revealed that UCMSC^S&XFM−CD^ and UCMSC^SCM^ showed high expression of CD29, CD44, CD73, CD90 and CD105, but lacked expression of CD14, CD19, CD34, CD45 and HLA-DR surface antigens. **C** Representative images of osteogenic, chondrogenic, and adipogenic differentiation of UCMSC^S&XFM−CD^ and UCMSC^SCM^ after specific inductions and staining. Scale bars = 100 μm

While both UCMSC^S&XFM−CD^ and UCMSC^SCM^ exhibited the essential fibroblast-like structure of MSCs, UCMSC^S&XFM−CD^ appeared slender and bright (Fig. 1A). As shown by flow cytometry, both cell types showed high levels (> 95%) of CD29, CD44, CD73, CD90, and CD105 but poor expression (< 2%) of the CD14, CD19, CD34, CD45, and HLA-DR surface antigens (Fig. 1B). The abilities of UCMSC^S&XFM−CD^ and UCMSC^SCM^ to differentiate into osteocytes, chondrocytes, and adipocytes were confirmed by Alizarin Red, Alcian Blue, and Oil Red-O staining, respectively (Fig. 1C).

### Toxicity evaluation of UCMSCS^&XFM−CD^ and UCMSC^SCM^

**Fig. 2:**
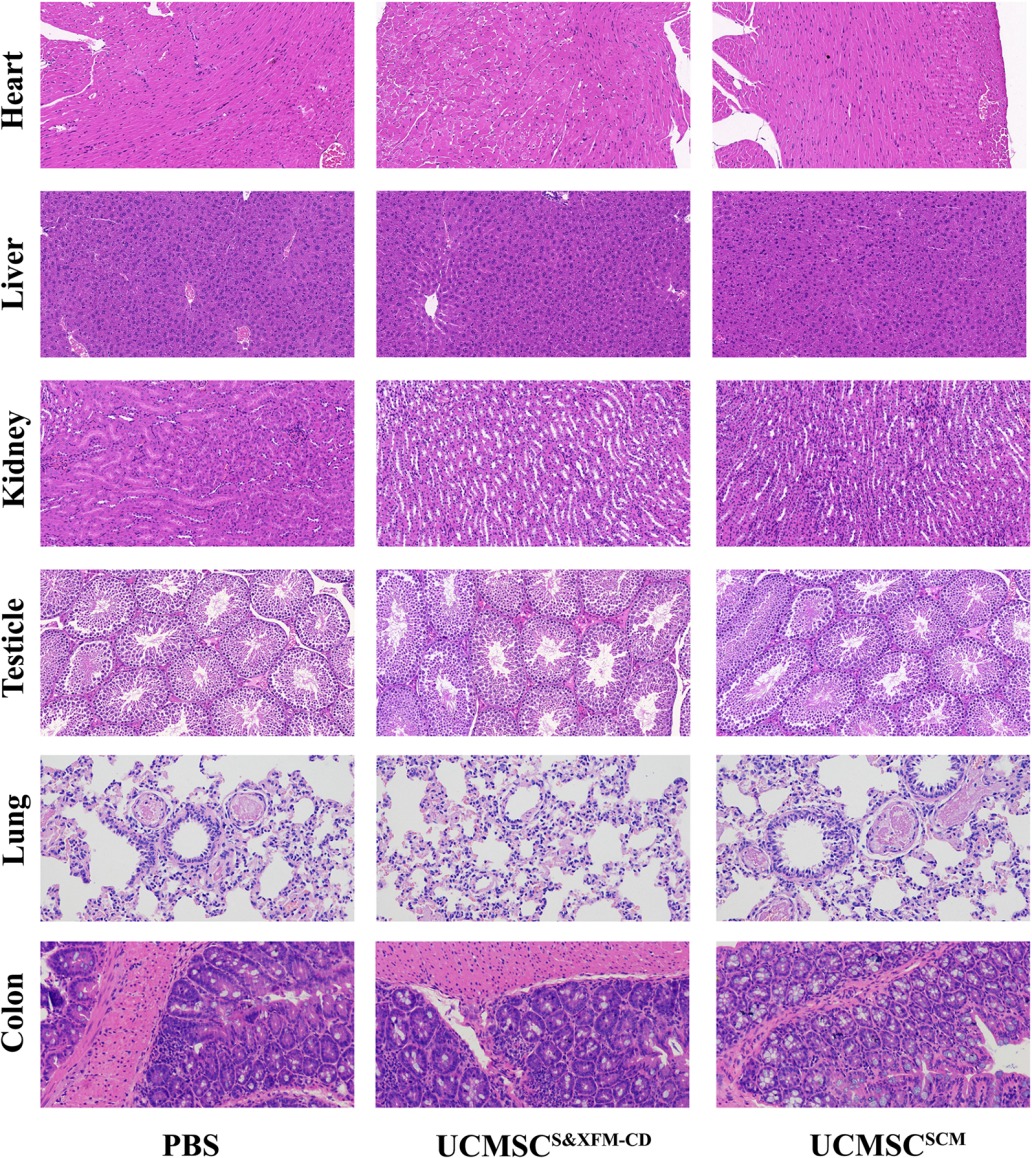
Toxicity evaluation of UCMSC^S&XFM−CD^ and UCMSC^SCM^. H&E-stained lung, liver, kidney, heart, testicle and colon in PBS, UCMSC^S&XFM−CD^ or UCMSC^SCM^ group for 28 days. Images were captured at magnification of 10×

To evaluate potential toxic effects, rats received intraperitoneal injections of UCMSC^SCM^, UCMSC^S&XFM−CD^, or PBS followed by assessments on days 14 and 35. The rats in all three groups survived. The vital signs, including body weight, temperature, heart rate, and blood pressure (both systolic and diastolic) of rats in the UCMSC^SCM^ and UCMSC^S&XFM−CD^ groups were not significantly different from those in the PBS group at days 14 and 35 after cell transplantation (Table 1). Compared with the PBS group, alkaline phosphatase levels were significantly increased in the UCMSC^SCM^ group on the 14th day (*P* < 0.05), but no changes were observed in the UCMSC^S&XFM−CD^ group (Table 2). Albumin, urea nitrogen, and glucose in both the UCMSC^SCM^ and UCMSC^SCM^ groups were significantly increased (all *P* < 0.05), while there were no significant changes in other blood biochemical indices (Table 2). On day 35, none of the blood biochemical indices in the UCMSC^S&XFM−CD^ and UCMSC^SCM^ groups differed significantly from those in the PBS group (Table 2). Microscopic diagnosis showed no obvious histopathological changes associated with MSCs in the six tissues analyzed (lung, liver, kidney, heart, testis, and colon) in the UCMSC^S&XFM−CD^ and UCMSC^SCM^ groups (Fig. 2).

**Table 2:** Biochemical indicators test

Parameters	Day 14			Day 35		
	PBS	UCMSC^SCM^	UCMSC^S&XFM−CD^	PBS	UCMSC^SCM^	UCMSC^S&XFM−CD^
ALT (U·L^−1^)	33.36 ± 6.24	34.64 ± 5.56	33.44 ± 6.19	33.50 ± 5.95	29.22 ± 5.05	28.70 ± 6.08
AST (U·L^−1^)	86.70 ± 15.78	88.38 ± 16.28	97.55 ± 16.57	86.39 ± 19.1	90.93 ± 15.19	84.28 ± 16.16
ALP (U·L^−1^)	103.04 ± 12.41	130.27 ± 24.65*	123.38 ± 29.76	95.12 ± 7.22	97.53 ± 19.1	92.01 ± 11.6
TBIL (µmol·L^−1^)	4.19 ± 1.23	5.27 ± 1.51	5.36 ± 1.32	4.1 ± 1.27	97.53 ± 19.1	5.04 ± 1.21
TP (mg·L^−1^)	494.8 ± 12.28	486.13 ± 11.92	485.77 ± 14.12	483.28 ± 10.95	482.57 ± 13.25	482.53 ± 22.48
ALB (g·L^−1^)	30.38 ± 2.65	38.45 ± 1.26*	34.48 ± 2.35*	32.01 ± 3.76	35.05 ± 1.98	33.22 ± 1.87
BUN (mmol·L^−1^)	10.17 ± 0.57	11.47 ± 1.27*	11.31 ± 1.18*	9.68 ± 0.64	10.31 ± 0.97	9.58 ± 0.88
Cr (µmol·L^−1^)	19.33 ± 3.58	19.65 ± 1.59	16.51 ± 2.32	18.09 ± 3.77	19.23 ± 3.45	20.05 ± 3.31
TG (mmol·L^−1^)	2.29 ± 0.69	2.44 ± 0.43	3.05 ± 0.25	2.41 ± 0.53	2.41 ± 0.58	2.78 ± 0.55
TC (mmol·L^−1^)	2.06 ± 0.23	2.12 ± 0.24	2.3 ± 0.34	2.1 ± 0.36	2.39 ± 0.34	2.21 ± 0.2
GLU (mmol·L^−1^)	3.69 ± 0.73	4.86 ± 0.46*	4.62 ± 0.6*	4.47 ± 0.68	4.81 ± 0.43	4.91 ± 0.19

Data are expressed as mean ± SD

*ALT* alanine transaminase, *AST* aspartate aminotransferase, *ALP* alkaline phosphatase, *TBIL* total bilirubin, *TP* total protein, *ALB* albumin, *BUN* blood urea nitrogen, *Cr* crea, *TG* triglyceride, *TC* total cholesterol, *GLU* glucose

*P < 0.05 vs PBS group

### Immunogenicity of UCMSCS^&XFM−CD^ and UCMSC^SCM^

No significant changes were observed in the routine blood measurements between the UCMSC^SCM^ and UCMSC^S&XFM−CD^ groups compared with the PBS group on days 14 and 35 (Table 3). The CD3^+^, CD4^+^, and CD8^+^ T lymphocyte proportions and CD4^+^/CD8^+^ ratios in the UCMSC^S&XFM−CD^ group were not significantly different from those in the UCMSC^SCM^ group (Table 4). One rat was found to be positive for serum IFN-γ in the UCMSC^SCM^ group but no IFN-γ positivity was observed in the PBS and UCMSC^S&XFM−CD^ groups. All rats were negative for IL-4 and IL-10, and the serum TNF-α level did not change in the UCMSC^SCM^ group relative to the PBS group (Table 5). All rats were evaluated for the production of antibodies binding to BSA and apoB-100. As expected, no antibodies were detected in the UCMSC^S&XFM−CD^ group on days 21, 28, and 35. While in the UCMSC^SCM^ group, one, three and four rats had developed antibody against BSA on days 21, 28, and 35, and one, two and three rats for antibody against apoB-100, respectively (Table 6).

**Table 3:** Blood routine test

Parameters	Day 14			Day 35		
	PBS	UCMSC^SCM^	UCMSC^S&XFM−CD^	PBS	UCMSC^SCM^	UCMSC^S&XFM−CD^
RBC (× 10^12^⋅L-1)	7.12 ± 0.62	7.02 ± 0.32	7.15 ± 0.41	7.38 ± 0.98	7.42 ± 0.79	7.82 ± 0.96
WBC (× 10^9^⋅L-1)	6.91 ± 2.16	6.25 ± 1.87	7.18 ± 1.74	7.19 ± 1.51	6.81 ± 1.42	7.25 ± 1.34
PLT (× 10^9^⋅L-1)	930.50 ± 111.5	1001.88 ± 66.35	860.13 ± 66.88	995.13 ± 88.49	10175.50 ± 65.90	992.13 ± 139.39
Hb (g⋅L-1)	135.50 ± 12.82	136.50 ± 7.03	135.50 ± 5.24	126.13 ± 7.62	128.25 ± 8.05	129.88 ± 7.36
NEUTRO (× 10^9^⋅L-1)	0.59 ± 0.16	0.61 ± 0.18	0.65 ± 0.26	0.75 ± 0.36	0.75 ± 0.36	0.65 ± 0.31
LYM (× 10^9^⋅L-1)	4.79 ± 1.59	4.06 ± 0.40	6.46 ± 2.44	7.40 ± 2.01	6.90 ± 2.10	6.99 ± 2.10

Data are expressed as mean ± SD

*RBC* red blood cells, *WBC* white blood cells, *PLT* platelets, *Hb* haemoglobin, *NEUTRO* neutrophils, *LYM* lymphocyte

Compared with the PBS group, *P* ≥ 0.05, compared with the UCMSC^SCM^ group, *P* ≥ 0.05

**Table 4:** The sub-population of T-cells

Parameters	Day 14			Day 35		
	PBS	UCMSC^SCM^	UCMSC^S&XFM−CD^	PBS	UCMSC^SCM^	UCMSC^S&XFM−CD^
CD3^+^	66.39 ± 7.97	68.33 ± 9.05	70.21 ± 10.69	67.48 ± 11.07	68.51 ± 9.34	69.65 ± 8.97
CD3^+^CD4^+^	35.42 ± 5.52	33.14 ± 4.08	34.26 ± 2.99	36.35 ± 6.85	34.32 ± 7.04	37.15 ± 4.07
CD3^+^CD8^+^	20.37 ± 3.64	24.61 ± 7.61	22.74 ± 5.06	22.08 ± 4.02	23.09 ± 5.72	21.84 ± 4.48
CD4/CD8	1.74 ± 0.52	1.35 ± 0.54	1.5 ± 0.59	1.65 ± 0.70	1.48 ± 0.23	1.70 ± 0.91

Data are expressed as mean ± SD; Compared with the PBS group, *P* ≥ 0.05, compared with the UCMSC^SCM^ group, *P* ≥ 0.05

**Table 5:** Cytokine levels

Parameters	Day 14			Day 35		
	PBS	UCMSC^SCM^	UCMSC^S&XFM−CD^	PBS	UCMSC^SCM^	UCMSC^S&XFM−CD^
IFN-γ (pg/ml)	Not detected	Not detected*	Not detected	Not detected	Not detected	Not detected
TNF-α (pg/ml)	5.15 ± 2.23	7.86 ± 3.09	6.74 ± 2.38	4.93 ± 3.09	7.64 ± 2.67	6.62 ± 2.48
IL-4 (pg/ml)	Not detected	Not detected	Not detected	Not detected	Not detected	Not detected
IL-10 (pg/ml)	Not detected	Not detected	Not detected	Not detected	Not detected	Not detected

Data are expressed as mean ± SD

*One mouse which was positive for IFN-γ in the UCMSC^SCM^ group. Compared with the PBS group,* P* ≥ 0.05, compared with the UCMSC^SCM^ group,* P* ≥ 0.05

**Table 6:** Incidence of antibody to BSA and apoB-100

Parameters	Day 21		Day 28		Day 35	
	UCMSC^SCM^	UCMSC^S&XFM−CD^	UCMSC^SCM^	UCMSC^S&XFM−CD^	UCMSC^SCM^	UCMSC^S&XFM−CD^
Anti-BSA	1/5	0/5	3/5	0/5	4/5	0/5
Anti-apoB-100	1/5	0/5	2/5	0/5	3/5	0/5

The incidence of antibody was presented by the no. of rats which were positive for antibody/the no. of rats which were measured in all

### Biodistribution of UCMSCS^&XFM−CD^ and UCMSC^SCM^

**Fig. 3:**
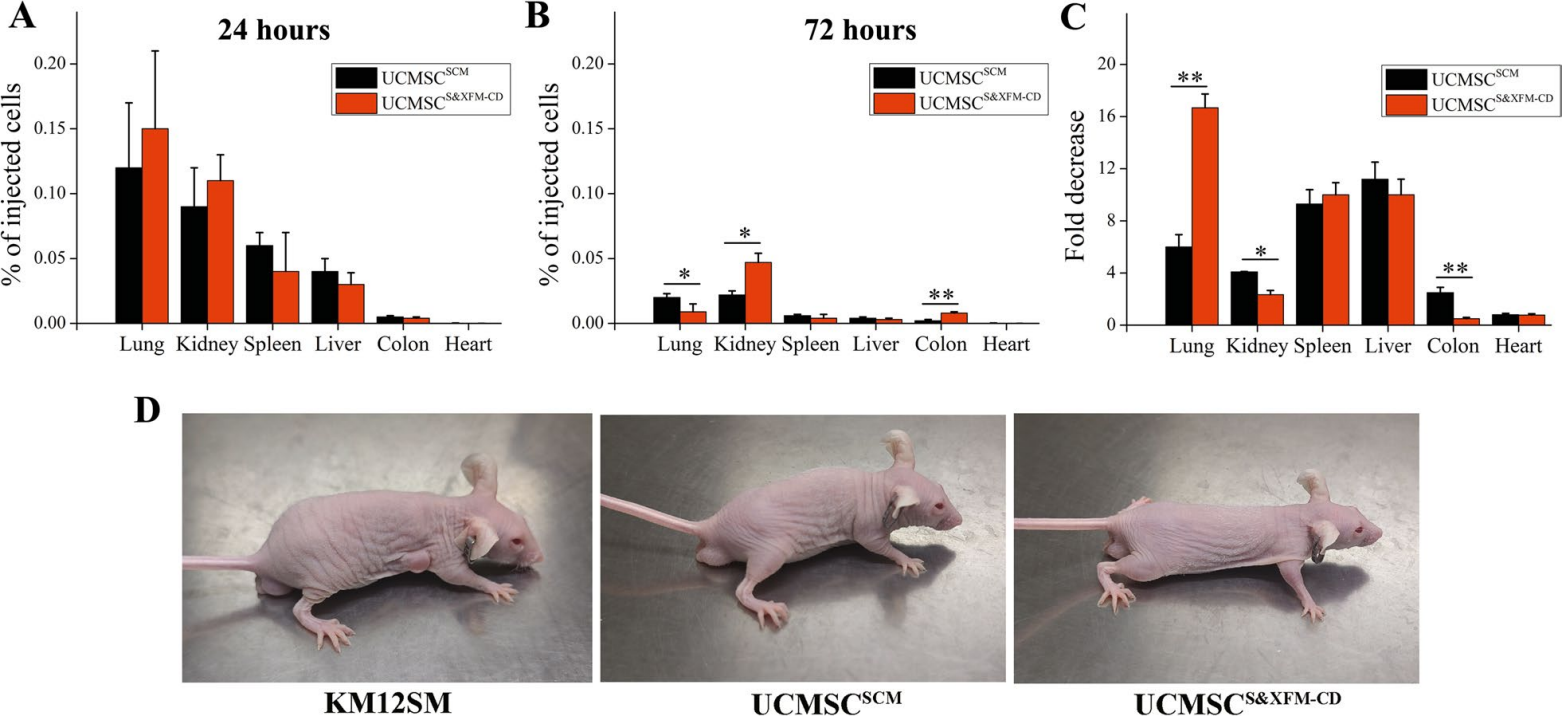
Migration and tumorigenic potential of UCMSC^S&XFM−CD^ and UCMSC^SCM^. Percentages of UCMSC^SCM^ or UCMSC^S&XFM−CD^ in lung, kidney, liver, testicle, heart, and colon at 24 (**A**) and 72 h (**B**) after intraperitoneally injection. **C** The fold decrease in the number of UCMSC^S&XFM−CD^ and UCMSC^SCM^ in each organ from 24 to 72 h. **D** Representative images of tumorigenic activity of UCMSC^S&XFM−CD^ and UCMSC^SCM^

Previously, we found that intraperitoneal injection of both UCMSC^S&XFM−CD^ and UCMSC^SCM^ resulted in minimal recruitment and persistence of the cells in the colon after 72 h, shown by fluorescence imaging [15]. For further tracking and quantification of UCMSCs, UCMSC^S&XFM−CD^, and UCMSC^SCM^ (2 × 10^6^ cells) after intraperitoneal injection, the lungs, kidneys, livers, testes, hearts, and colons of the mice were collected at 24 and 72 h after transplantation and were quantified by qRT-PCR measurements and the construction of standard curves, as described previously [22]. Both UCMSC^S&XFM−CD^ and UCMSC^SCM^ were observed in the hearts, lungs, livers, testes, kidneys, and colons, and a large number of injected cells were blocked in the lungs at 24 h after intraperitoneal injection in both groups (Fig. 3A). Then, the number of UCMSC^S&XFM−CD^ blocked in the lungs quickly decreased, and significantly fewer UCMSC^S&XFM−CD^ than UCMSC^SCM^ were in the lungs at 72 h after injection (*P* < 0.05, Fig. 3B, C). However, more UCMSC^S&XFM−CD^ than UCMSC^SCM^ retained in the kidneys and colon (both *P* < 0.05–0.01, Fig. 3B, C).

### Tumorigenic potential of UCMSCS^&XFM−CD^ and UCMSC^SCM^

Mice inoculated with the KM12SM cell line formed progressive nodules at the injection site, which were found after sacrifice to cover a macroscopic tumor. The mice treated with UCMSC^SCM^, UCMSC^S&XFM−CD^, or PBS showed no clinical signs during the experimental period (Fig. 3D).

### Tumor-promoting effect of UCMSCS^&XFM−CD^ and UCMSC^SCM^

**Fig. 4:**
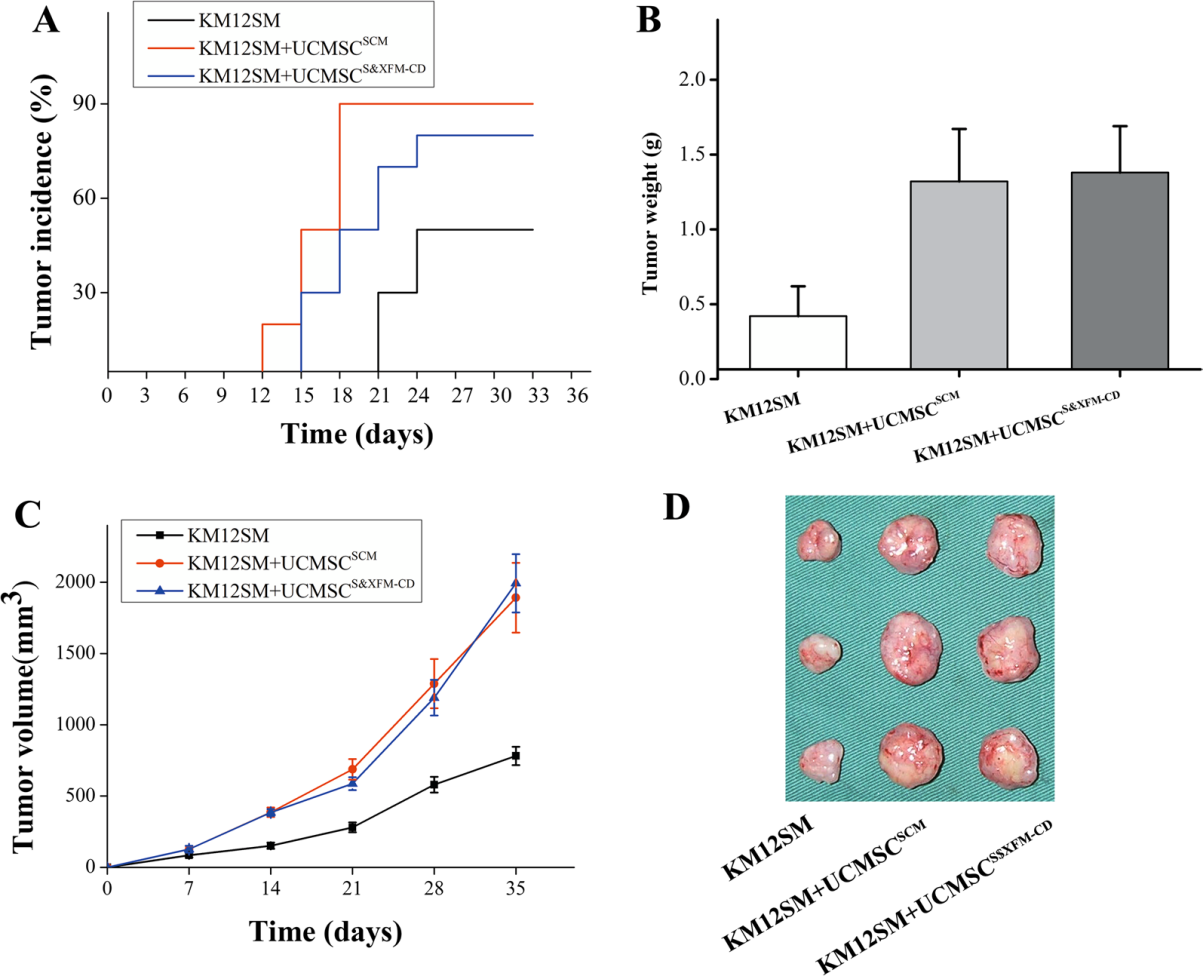
Tumor-promoting effect of UCMSC^S&XFM−CD^ and UCMSC^SCM^. **A** Kaplan–Meier curve. The weights **B** and volumes **C** of the excised tumors between the UCMSC^S&XFM−CD^ and UCMSC^SCM^ groups. **D** Representative images of the excised tumors between the UCMSC^S&XFM−CD^ and UCMSC^SCM^ groups

We next investigated the role of UCMSC^S&XFM−CD^ and UCMSC^SCM^ on tumor growth in nude mice. Subcutaneous injections of 2 × 10^6^ KM12SM cells were given into the right flanks of the mice, together with equal numbers of UCMSC^S&XFM−CD^ or UCMSC^SCM^. The control group was injected with PBS only. Co-injection of KM12SM cells with UCMSC^SCM^ reduced the time taken for tumor formation to 13 days compared with 15 days in the group co-injected with UCMSC^S&XFM−CD^ and 21 days in the PBS group (Fig. 4A). UCMSC^SCM^ also increased the incidence of tumor development in the mice to 90% compared with 80% in mice co-injected with UCMSC^S&XFM−CD^ and 50% in the PBS group (Fig. 4A). However, neither the weights nor volumes of the tumors differed significantly between the UCMSC^S&XFM−CD^ and UCMSC^SCM^ groups (Fig. 4B–D).

## Discussion

The evaluation of the safety of UCMSCs, including assessment of toxicity, abnormal immunological reactions, biodistribution, and tumorigenicity, is a major focus in the development of “cell-based therapies” [23]. UCMSCs cultured in FBS or platelet lysates have been increasingly shown to be safe without deterioration [24–26]. However, the usage of FBS or platelet lysates makes standardization of cell manufacturing process difficult because of the poorly defined nature. The use of S&XFM–CD, as an ideal cell culture medium for cell therapy, has been proposed to replace SCM during the preparation of stem cells [10]. In this study, we demonstrated that intraperitoneally transplanted UCMSC^S&XFM−CD^ did not cause significant changes in general vital signs, histopathology, routine blood indices, T lymphocyte subsets, and serum cytokine levels, and showed no obvious tumorigenic activity in experimental rats. Furthermore, UCMSC^S&XFM−CD^ did not result in the production of anti-BSA and apoB-100 antibodies, in contrast to UCMSC^SCM^ which has a very high chance of producing antibodies to BSA (80%) and apoB-100 (60%). In addition, UCMSC^S&XFM−CD^ extended the time taken for KM12SM cell tumor formation and decreased the tumor incidence compared with UCMSC^SCM^. Finally, intraperitoneally injected UCMSC^S&XFM−CD^ were less likely to be blocked by the lungs and migrated more easily to the kidneys and colon than UCMSC^SCM^ and had almost disappeared 72 h after transplantation. Thus, these findings indicate that UCMSC^S&XFM−CD^ displayed an improved safety performance.

Although the levels of albumin, urea nitrogen, and glucose sugar were significantly elevated in the UCMSC^S&XFM−CD^ group at day 14, the levels of these indicators are still within the reference range [27, 28]. According to the general guidelines for clinicopathological data analysis, clinicopathological parameters cannot be evaluated in isolation, and damage to organs is only likely when the activities or concentrations of these indicators are higher than twice the normal upper limit [28, 29]. Moreover, no significant differences were shown between the UCMSC^S&XFM−CD^ and UCMSC^SCM^ group, suggesting that the changes in blood biochemical indices caused by the intraperitoneal administration of UCMSCs were independent of the cell culture conditions. In addition, the levels of these three indicators did not differ significantly between the UCMSC^S&XFM−CD^ and PBS groups on day 35. This indicates that changes in blood biochemical indices resulting from transplantation of UCMSCs into the abdominal cavity occur over a period of time, and that the indicators will gradually return to normal over time.

Intraperitoneally administered UCMSC^S&XFM−CD^ did not induce immune changes, including alterations in the levels of routine blood indices, inflammatory cytokines, and T-cell sub-populations, which is consistent with the low expression of HLA-DR in UCMSC^S&XFM−CD^ [13]. This suggested low immunogenicity of the allogeneic UCMSCs in S&XFM–CD, as they did not cause alloimmune reactions. Alloantibodies can also result from exposure to FBS [8, 30] and MSCs are often grown in medium containing FBS. Uptake of FBS by MSCs has been observed, together with the presence of FBS components on the cell surfaces [31]. It has also been reported that anti-BSA antibodies interfered with the efficacy of treatment [32] and an animal study observed marked humoral responses after administration of MSCs cultured in 20% FBS [31]. UCMSC^SCM^ thus have a very high chance of inducing antibody production, while no antibodies are produced in UCMSC^S&XFM−CD^ due to the absence of animal-derived ingredients. This is easy to understand as serum-free media have no animal-derived components. This feature of UCMSC^S&XFM−CD^ is thus a great advantage in clinical applications, especially for repeat infusions.

The in vivo distribution of MSCs is closely related to the transplantation route [33]. The distribution of MSCs after intravenous infusion has been reported in many studies [34, 35]. A previous study confirmed that delivery by intraperitoneal injection resulted in greater MSC distribution and better recovery from experimental colitis than either intravenous or anal injections [14], and intraperitoneally administered UCMSC^S&XFM−CD^ showed greater therapeutic efficacy than UCMSC^SCM^. Interestingly, we found that intraperitoneal infusion resulted in a similar cell distribution to intravenous infusion, such as lung preference within 24 h, but changing to a gradual predominance of kidney and colon distribution at 48 h [36]. We found that intraperitoneally injected UCMSC^S&XFM−CD^ are less likely to be blocked by the lungs, which might reduce the risk of vascular embolism. The lung is the organ in which the injected cells are blocked most heavily compared with other organs and pulmonary complications are more likely to be life-threatening. This is related to the size of the cells, consistent with the findings of previous studies [24]. However, it is inconsistent with low expression of SDF-1 in UCMSC^S&XFM−CD^ [13, 37], suggesting that cell size plays a greater role than SDF-1 expression. Of course, other unknown factors cannot be ruled out. In addition, UCMSC^S&XFM−CD^ migrated more easily to the colon than UCMSC^SCM^, which may be related to the intraperitoneal injection route. This may be more beneficial for colitis. Although more UCMSC^S&XFM−CD^ migrated to the kidneys in our study, in view of the differences about cell homing in healthy and colitis rats, more experiments are still needed to further confirm the distribution of cells in colitis rats. In this study, we used normal rats with normal immune function, while most of the patients in the clinic would have abnormal immune function, especially immune-related diseases. Thus, the life span and migration rate of UCMSCs may differ between the normal and injured colon due to the inflammatory microenvironment, and the secretion of inflammatory factors may attract UCMSCs homing to and staying in the injury area, which is supported by our previous findings [15].

Although there is still controversy surrounding the ability of MSCs to induce tumors, some studies have indicated that MSCs can indeed induce both tumor development and metastasis [38, 39]. The developers of cell therapy products are required to conduct tumor-promoting tests and consult with regulators. Our results show that UCMSC^S&XFM−CD^ have a lower tumor-promoting effect than UCMSC^SCM^. We speculate that this may be due to the reduced release of SDF-1 by UCMSC^S&XFM−CD^ [13, 40]. However, our previous results showed that UCMSC^S&XFM−CD^ expressed high levels of PDGF and IGF-1 [13], and it has been found that these cytokines enhance MSC-mediated tumor promotion [41, 42]. This finding conflicts with our results and may be the consequence of different cell sources [43], different culture conditions, or different tumors [44, 45]. In short, UCMSC^S&XFM−CD^ have low tumor-promoting ability, which would likely increase the use of cells. However, further research on the exact mechanism underlying the reduced tumor-promoting effect is needed.

## Conclusions

In conclusion, the present study provided evidence that UCMSC^S&XFM−CD^ display an improved safety performance. In combination with our previous reports that UCMSC^S&XFM−CD^ are more effective in promoting recovery from experimental colitis [13, 15], we encourage the use of UCMSC^S&XFM−CD^ in future clinical trials.

### Supplementary Information

**Additional file 1**. **Table S1**: Flow cytometry antibodies from BD bioscience.

## Data Availability

The data presented in this study are available on request from the corresponding author.

## Abbreviations

UC: Umbilical cord
MSCs: Mesenchymal stromal cells
UCMSCs: UC-derived MSCs
FBS: Fetal bovine serum
SCM: Serum-containing media
S&XFM–CD: Serum-free, xeno-free, and chemically defined medium
PBS: Phosphate-buffered saline
SD: Sprague–Dawley
BSA: Bovine serum albumin
apoB-100: Apolipoprotein B-100
IFN: Interferon
TNF: Tumor necrosis factor
IL: Interleukin

## References

[1] Najar M, Melki R, Khalife F, Lagneaux L, Bouhtit F, Moussa Agha D, Fahmi H, Lewalle P, Fayyad-Kazan M, Merim M (2021). Therapeutic mesenchymal stem/stromal cells: value, challenges and optimization. Front Cell Dev Biol.

[2] Viswanathan S, Shi Y, Galipeau J, Krampera M, Leblanc K, Martin I, Nolta J, Phinney DG, Sensebe L (2019). Mesenchymal stem versus stromal cells: international society for cell & gene therapy (isct(r)) mesenchymal stromal cell committee position statement on nomenclature. Cytotherapy.

[3] Raileanu VN, Whiteley J, Chow T, Kollara A, Mohamed A, Keating A, Rogers IM (2019). Banking mesenchymal stromal cells from umbilical cord tissue: large sample size analysis reveals consistency between donors. Stem Cells Transl Med.

[4] Kabat M, Bobkov I, Kumar S, Grumet M (2019). Trends in mesenchymal stem cell clinical trials 2004–2018: is efficacy optimal in a narrow dose range?. Stem Cells Transl Med.

[5] Merimi M, Fahmi H, De Kock J, Beguin C, Burny A, Moll G, Poggi A, Najar M (2022). Mesenchymal stem/stromal cells as a therapeutic tool in cell-based therapy and regenerative medicine: an introduction expertise to the topical collection. Cells.

[6] Phinney DG, Galipeau J (2019). C. Msc Committee Of The International Society Of, and T Gene, Manufacturing mesenchymal stromal cells for clinical applications: a survey of Good Manufacturing Practices at U.S. academic centers. Cytotherapy.

[7] Solomon J, Csontos L, Clarke D, Bonyhadi M, Zylberberg C, McNiece I, Kurtzberg J, Bell R, Deans R (2016). Current perspectives on the use of ancillary materials for the manufacture of cellular therapies. Cytotherapy.

[8] Owens SD, Kol A, Walker NJ, Borjesson DL (2016). Allogeneic mesenchymal stem cell treatment induces specific alloantibodies in horses. Stem Cells Int.

[9] van der Valk J, Bieback K, Buta C, Cochrane B, Dirks WG, Fu J, Hickman JJ, Hohensee C, Kolar R, Liebsch M, Pistollato F, Schulz M, Thieme D, Weber T, Wiest J, Winkler S, Gstraunthaler G (2018). Fetal bovine serum (FBS): past - present - future. Altex.

[10] Nguyen LT, Tran NT, Than UTT, Nguyen MQ, Tran AM, Do PTX, Chu TT, Nguyen TD, Bui AV, Ngo TA, Hoang VT, Hoang NTM (2022). Optimization of human umbilical cord blood-derived mesenchymal stem cell isolation and culture methods in serum- and xeno-free conditions. Stem Cell Res Ther.

[11] Cao J, Hao J, Wang L, Tan Y, Tian Y, Li S, Ma A, Fu B, Dai J, Zhai P, Xiang P, Zhang Y, Cheng T, Peng Y, Zhou Q, Zhao T (2021). Developing standards to support the clinical translation of stem cells. Stem Cells Transl Med.

[12] Zhao Q, Han Z, Wang J, Han Z (2021). Development and investigational new drug application of mesenchymal stem/stromal cells products in China. Stem Cells Transl Med.

[13] Wu X, Ma Z, Wu D (2020). Derivation of clinical-grade mesenchymal stromal cells from umbilical cord under chemically defined culture condition - platform for future clinical application. Cytotherapy.

[14] Wang M, Liang C, Hu H, Zhou L, Xu B, Wang X, Han Y, Nie Y, Jia S, Liang J, Wu K (2016). Intraperitoneal injection (IP), Intravenous injection (IV) or anal injection (AI)? Best way for mesenchymal stem cells transplantation for colitis. Sci Rep.

[15] Wu X, Wu D, Mu Y, Zhao Y, Ma Z (2020). Serum-free medium enhances the therapeutic effects of umbilical cord mesenchymal stromal cells on a murine model for acute colitis. Front Bioeng Biotechnol.

[16] Lindblad RW, Ibenana L, Wagner JE, McKenna DH, Hei DJ, Hematti P, Couture LA, Silberstein LE, Armant M, Rooney CM, Gee AP, Welniak LA, Heath Mondoro T, Wood DA, Styers D (2015). Cell therapy product administration and safety: data capture and analysis from the Production assistance for cellular therapies (PACT) program. Transfusion.

[17] Toyserkani NM, Jorgensen MG, Tabatabaeifar S, Jensen CH, Sheikh SP, Sorensen JA (2017). Concise review: a safety assessment of adipose-derived cell therapy in clinical trials: a systematic review of reported adverse events. Stem Cells Transl Med.

[18] Egger D, Lavrentieva A, Kugelmeier P, Kasper C (2022). Physiologic isolation and expansion of human mesenchymal stem/stromal cells for manufacturing of cell-based therapy products. Eng Life Sci.

[19] Wu X, Kang H, Liu X, Gao J, Zhao K, Ma Z (2016). Serum and xeno-free, chemically defined, no-plate-coating-based culture system for mesenchymal stromal cells from the umbilical cord. Cell Prolif.

[20] Wu X, Mu Y, Yao J, Lin F, Wu D, Ma Z (2022). Adipose-derived stem cells from patients with ulcerative colitis exhibit impaired immunosuppressive function. Front Cell Dev Biol.

[21] Sakamoto N, Tsuji K, Muul LM, Lawler AM, Petricoin EF, Candotti F, Metcalf JA, Tavel JA, Lane HC, Urba WJ, Fox BA, Varki A, Lunney JK, Rosenberg AS (2007). Bovine apolipoprotein B-100 is a dominant immunogen in therapeutic cell populations cultured in fetal calf serum in mice and humans. Blood.

[22] Song WJ, Li Q, Ryu MO, Ahn JO, Ha Bhang D, Chan Jung Y, Youn HY (2017). TSG-6 secreted by human adipose tissue-derived mesenchymal stem cells ameliorates DSS-induced colitis by inducing M2 macrophage polarization in mice. Sci Rep.

[23] Zhuang WZ, Lin YH, Su LJ, Wu MS, Jeng HY, Chang HC, Huang YH, Ling TY (2021). Mesenchymal stem/stromal cell-based therapy: mechanism, systemic safety and biodistribution for precision clinical applications. J Biomed Sci.

[24] Liang Q, Li Q, Ren B, Yin ZQ (2022). Intravenous infusion of small umbilical cord mesenchymal stem cells could enhance safety and delay retinal degeneration in RCS rats. BMC Ophthalmol.

[25] Shi L, Zhang Y, Dong X, Pan Y, Ying H, Chen J, Yang W, Zhang Y, Fei H, Liu X, Wei C, Lin H, Zhou H, Zhao C, Yang A, Zhou F, Zhang S (2022). Toxicity from a single injection of human umbilical cord mesenchymal stem cells into rat ovaries. Reprod Toxicol.

[26] Li X, Huang Q, Zhang X, Xie C, Liu M, Yuan Y, Feng J, Xing H, Ru L, Yuan Z, Xu Z, Yang Y, Long Y, Xing C, Song J, Hu X, Xu Q (2022). Reproductive and developmental toxicity assessment of human umbilical cord mesenchymal stem cells in rats. Front Cell Dev Biol.

[27] R.L. Hall, Clinical pathology of laboratory animals, 2015.

[28] Dirven H, Vist GE, Bandhakavi S, Mehta J, Fitch SE, Pound P, Ram R, Kincaid B, Leenaars CHC, Chen M, Wright RA, Tsaioun K (2021). Performance of preclinical models in predicting drug-induced liver injury in humans: a systematic review. Sci Rep.

[29] Tayebi B, Babaahmadi M, Pakzad M, Hajinasrollah M, Mostafaei F, Jahangiri S, Kamali A, Baharvand H, Baghaban Eslaminejad M, Hassani SN, Hajizadeh-Saffar E (2022). Standard toxicity study of clinical-grade allogeneic human bone marrow-derived clonal mesenchymal stromal cells. Stem Cell Res Therapy.

[30] Sundin M, Ringden O, Sundberg B, Nava S, Gotherstrom C, Le Blanc K (2007). No alloantibodies against mesenchymal stromal cells, but presence of anti-fetal calf serum antibodies, after transplantation in allogeneic hematopoietic stem cell recipients. Haematologica.

[31] Spees JL (2004). Internalized antigens must be removed to prepare hypoimmunogenic mesenchymal stem cells for cell and gene therapy. Mol Therapy J Am Soc Gene Therapy.

[32] Horwitz EM, Gordon PL, Koo WK, Marx JC, Neel MD, McNall RY, Muul L, Hofmann T (2002). Isolated allogeneic bone marrow-derived mesenchymal cells engraft and stimulate growth in children with osteogenesis imperfecta: Implications for cell therapy of bone. Proc Natl Acad Sci USA.

[33] Kamiyama Y, Naritomi Y, Moriya Y, Yamamoto S, Kitahashi T, Maekawa T, Yahata M, Hanada T, Uchiyama A, Noumaru A, Koga Y, Higuchi T, Ito M, Komatsu H, Miyoshi S, Kimura S, Umeda N, Fujita E, Tanaka N, Sugita T, Takayama S, Kurogi A, Yasuda S, Sato Y (2021). Biodistribution studies for cell therapy products: current status and issues. Regen Ther.

[34] Srinivasan RC, Kannisto K, Strom SC, Gramignoli R (2019). Evaluation of different routes of administration and biodistribution of human amnion epithelial cells in mice. Cytotherapy.

[35] Brooks A, Futrega K, Liang X, Hu X, Liu X, Crawford DHG, Doran MR, Roberts MS, Wang H (2018). Concise review: quantitative detection and modeling the in vivo kinetics of therapeutic mesenchymal stem/stromal cells. Stem Cells Transl Med.

[36] Aguilera Y, Mellado-Damas N, Olmedo-Moreno L, Lopez V, Panadero-Moron C, Benito M, Guerrero-Cazares H, Marquez-Vega C, Martin-Montalvo A, Capilla-Gonzalez V (2021). Preclinical safety evaluation of intranasally delivered human mesenchymal stem cells in juvenile mice. Cancers.

[37] Luo Q, Zhang B, Kuang D, Song G (2016). Role of stromal-derived factor-1 in mesenchymal stem cell paracrine-mediated tissue repair. Curr Stem Cell Res Ther.

[38] Lan T, Luo M, Wei X (2021). Mesenchymal stem/stromal cells in cancer therapy. J Hematol Oncol.

[39] Antoon R, Wang XH, Saleh AH, Warrington J, Hedley DW, Keating A (2022). Pancreatic cancer growth promoted by bone marrow mesenchymal stromal cell-derived IL-6 is reversed predominantly by IL-6 blockade. Cytotherapy.

[40] Pan C, Fang Q, Liu P, Ma D, Cao S, Zhang L, Chen Q, Hu T, Wang J (2021). Mesenchymal stem cells with cancer-associated fibroblast-like phenotype stimulate SDF-1/CXCR4 axis to enhance the growth and invasion of b-cell acute lymphoblastic leukemia cells through cell-to-cell communication. Front Cell Dev Biol.

[41] Li X, Fan Q, Peng X, Yang S, Wei S, Liu J, Yang L, Li H (2022). Mesenchymal/stromal stem cells: necessary factors in tumour progression. Cell Death Discov.

[42] Raghavan S, Snyder CS, Wang A, McLean K, Zamarin D, Buckanovich RJ, Mehta G (2020). Carcinoma-associated mesenchymal stem cells promote chemoresistance in ovarian cancer stem cells via PDGF signaling. Cancers.

[43] Kidd S, Spaeth E, Watson K, Burks J, Lu H, Klopp A, Andreeff M, Marini FC (2012). Origins of the tumor microenvironment: quantitative assessment of adipose-derived and bone marrow-derived stroma. PLoS ONE.

[44] Lee HY, Hong IS (2017). Double-edged sword of mesenchymal stem cells: Cancer-promoting versus therapeutic potential. Cancer Sci.

[45] Xuan X, Tian C, Zhao M, Sun Y, Huang C (2021). Mesenchymal stem cells in cancer progression and anticancer therapeutic resistance. Cancer Cell Int.

