# Supplementary material for: Umbilical cord mesenchymal stromal cells in serum-free defined medium display an improved safety profile
Source: Stem Cell Res Ther. 2023 Dec 12;14:360. doi: 10.1186/s13287-023-03604-0 (PMC10717764; doi:10.1186/s13287-023-03604-0)
Supplement: Supplementary file 1 — Additional file 1. Table S1: Flow cytometry antibodies from BD bioscience. [file 13287_2023_3604_MOESM1_ESM.docx]

**Supplementary Table 1**

Flow cytometry antibodies from BD bioscience

| Antibody | Clone | Catalog number |
| --- | --- | --- |
| APC Mouse Anti-Human CD29 | MAR4 | 559883 |
| APC Mouse Anti-Human CD44 | G44-26 | 559942 |
| PE Mouse Anti-Human CD73 | AD2 | 550257 |
| FITC Mouse Anti-Human CD90 | 5E10 | 555595 |
| PerCP-Cy™5.5 Mouse anti-Human CD105 | 266 | 560819 |
| APC Mouse Anti-Human CD14 | M5E2 | 561383 |
| APC Mouse Anti-Human CD19 | SJ25C1 | 560252 |
| PE Mouse Anti-Human CD34 | 581 | 555822 |
| FITC Mouse Anti-Human CD45 | HI30 | 561865 |
| APC Mouse Anti-Human HLA-DR | G46-6 | 560744 |
| FITC Mouse Anti-Rat CD3 | 1F4 | 561801 |
| PE-Cy 7 Mouse Anti-Rat CD4 | OX-35 | 561578 |
| PE Mouse Anti-Rat CD8a | OX-8 | 559976 |
| PerCP Mouse IgG1 κ Isotype Control | MOPC-21 | 559425 |
| PE Mouse IgG1, κ Isotype Control | MOPC-21 | 559320 |
| FITC Mouse IgG1, κ Isotype Control | MOPC-21 | 551954 |
| APC Mouse IgG1 κ Isotype Control | MOPC-21 | 554681 |
